# The role of communication in breast cancer screening: a qualitative study with Australian experts

**DOI:** 10.1186/s12885-015-1749-0

**Published:** 2015-10-19

**Authors:** Lisa M. Parker, Lucie Rychetnik, Stacy M. Carter

**Affiliations:** 1 Centre for Values, Ethics and the Law in Medicine (VELiM), Sydney School of Public Health, The University of Sydney, Medical Foundation Building, K 25 (92-94 Parramatta Road), Sydney, NSW 2006 Australia; 2 School of Medicine Sydney, The University of Notre Dame (Australia), 160 Oxford St, Darlinghurst, NSW 2010 Australia

**Keywords:** Breast cancer, Mass screening, Communication, Decision making, Ethics, Qualitative research, Mammography

## Abstract

**Background:**

One well-accepted strategy for optimising outcomes in mammographic breast cancer screening is to improve communication with women about screening. It is not always clear, however, what it is that communication should be expected to achieve, and why or how this is so. We investigated Australian experts’ opinions on breast screening communication. Our research questions were: 1 What are the views of Australian experts about communicating with consumers on breast screening? 2 How do experts reason about this topic?

**Methods:**

We used a qualitative methodology, interviewing 33 breast screening experts across Australia with recognisable influence in the Australian mammographic breast cancer screening setting. We used purposive and theoretical sampling to identify experts from different professional roles (including clinicians, program managers, policy makers, advocates and researchers) with a range of opinions about communication in breast screening.

**Results:**

Experts discussed the topic of communication with consumers by focusing on two main questions: how strongly to guide consumers’ breast cancer screening choices, and what to communicate about overdiagnosis. Each expert adopted one of three approaches to consumer communication depending on their views about these topics. We labelled these approaches: Be screened; Be screened and here’s why; Screening is available please consider whether it’s right for you. There was a similar level of support for all three approaches. Experts’ reasoning was grounded in how they conceived of and prioritised their underlying values including: delivering benefits, avoiding harms, delivering more benefits than harms, respecting autonomy and transparency.

**Conclusions:**

There is disagreement between experts regarding communication with breast screening consumers. Our study provides some insights into this persisting lack of consensus, highlighting the different meanings that experts give to values, and different ways that values are prioritised. We suggest that explicit discussion about ethical values might help to focus thinking, clarify concepts and promote consensus in policy around communication with consumers. More specifically, we suggest that decision-makers who are considering policy on screening communication should begin with identifying and agreeing on the specific values to be prioritised and use this to guide them in establishing what the communication aims will be and which communication strategy will achieve those aims.

**Electronic supplementary material:**

The online version of this article (doi:10.1186/s12885-015-1749-0) contains supplementary material, which is available to authorized users.

## Background

Mammographic breast screening opportunities and programs have been introduced in many high-income countries over the past three decades [1–3], with the expectation of achieving significant population breast cancer mortality reduction. This outcome was suggested by evidence from early randomised control trials (RCTs) and cohort studies [1, 2] and later backed up by further studies and multi-study reviews [4, 5]. In Australia the government provides free biennial screening mammography for all women over 40 years of age through its national program *BreastScreen Australia* [6]. The government actively encourages the regular participation of women aged 50–74 years, with promotional communications focusing on this target age range [7]. An important focus of breast screening research has been how to communicate effectively with women in order to achieve high screening participation rates and realise the mortality benefits described in the literature [8].

At the same time there has been growing interest in encouraging and supporting members of the public to be more informed about health matters, including screening, and more engaged in decisions about their own healthcare [9–13]. This is partly underpinned by a desire to respect the autonomy of patients and healthcare consumers [14–16] and partly for reasons such as engendering greater public satisfaction, more efficient use of healthcare services and possibly even better health outcomes for individuals and communities [17–19].

More recently, uncertainties about both benefits and harms of breast screening have emerged. The benefits may be less than first anticipated from the early studies. Meta-analyses of what is by now a substantial body of RCT evidence on mammographic breast screening provide different estimates of benefit, depending on which of the RCTs are considered to be of sufficiently high quality to include in the review [20, 21]. There are also suggestions that the RCT evidence may be out of date, with recent improvements in breast cancer treatment together with increased awareness about prompt symptomatic presentation leaving less room for screening to have a beneficial effect [22, 23]. At the same time, a growing body of research is contributing to concern about harms associated with breast screening, including cumulative false positive tests [22] and overdiagnosis (the diagnosis of non-progressive or slowly progressive breast cancer through screening, a diagnosis that does not produce a net benefit for the woman diagnosed) [24–27]. The amount and significance of overdiagnosis harm is particularly contentious [23]. There is concern about whether or not hearing about these uncertainties and harms will deter women from screening, and indeed recent RCT evidence does suggest that women who are more informed about overdiagnosis express a lower intention to screen [28]. A perceived tension has thus arisen between the aim of achieving high breast screening participation rates and the aim of enabling women to make informed choices about screening, with debate about whether communication with consumers should focus on maximising participation or on communicating to support citizen’s knowledge and autonomy [29].

Official government policy endorses shared decision making to achieve informed choice in healthcare generally and in screening more specifically [30, 31]. Many claim that informed choice is particularly pertinent to screening because it actively targets healthy people rather than sick people who are seeking help for symptoms. Others highlight the importance of informed choice in those screening programs for which evidence about outcomes is insufficient or controversial, or where benefits and harms are finely balanced such that individual values become relatively more important in guiding decisions about being screened [32, 33]. There have been concerns that government directives to facilitate informed choice are not being adequately followed within breast screening [29, 34–36], with international criticism of breast screening information pamphlets on the grounds that they withhold important information about possible harms of breast screening [37–40], and suggestions that consumers should be explicitly encouraged to make their own choice whether or not to attend screening [36, 41, 42]. Not all authors prioritise the target of achieving informed consumer choice in cancer screening. Some prefer to focus on achieving adequate uptake in order to realise screening benefits [43]. Others have concerns about the process or reasoning behind such a target. For example, some writers suggest that it may be unreasonable to expect even fully informed citizens to take on what they depict as the burdensome task of decision making for cancer screening: weighing up uncertain benefits and harms, about which experts disagree [44]. Others contend that requiring or encouraging informed citizen decision making about cancer screening may be unecessary since, arguably, justification for it may rely on an inapporpriately narrow version of autonomy [45]. According to this view, a respectful approach should accommodate citizens who wish to rely on others to guide or choose for them. Finally, some argue that providing citizens with enough information to make fully informed screening choices may be prohibitively time consuming [46].

It is well-recognised that there are differences of opinion amongst clinicians regarding the involvement of patients in decision making for clinical care [47] and it is known that there are differences in how frequently or enthusiastically primary care practitioners discuss mammographic breast screening with their patients [48–50]. The ongoing discussions in the academic literature about the aims and content of breast screening communication suggests there is also likely to be diversity of expert opinion about policies for consumer decision making in relation to breast screening [29]. We could find no empirical work that examined this topic and to fill this gap we investigated the opinions and priorities of influential Australian experts with respect to breast screening communication with consumers. Our research questions were:

- What are the views of Australian experts about what and how we should communicate with consumers about breast screening?
- How do experts reason about this topic and how does this explain the positions they take?

## Methods

### Methodology

Our study was part of a larger project exploring the social and ethical issues around cancer screening in Australia [51]. Data collection from experts in breast screening was undertaken as a sub-study in the project, and this paper reports on one component of the sub-study. Other components of the sub-study involving analysis of different aspects of the data set have been written up separately [52]. We used an empirical, qualitative methodology. The emerging research field of “empirical bioethics” uses empirical research methods alongside traditional theoretical ethics in the context of healthcare and other biological sciences. Empirical methods are used to study and describe an ethical issue; theory is used to varying degrees by different researchers to shape the empirical study and inform interpretation and discussion of findings. We situate ourselves close to the style of Frith [53], who combines empirical and theoretical ethics in a symbiotic relationship, arguing that each can and should, inform eachother [54]. We used sampling, data collection and analysis strategies that were best suited to the particular circumstances and aims of our project, and enabled us to conduct our study with internal coherence [55–58].

### Participants and sampling

We sought to include influential breast screening experts from within Australia as participants. We defined influential experts as people with experience of working in a field directly related to breast screening in Australia and who had influence through one or more of: senior service delivery; academic or lay publication; membership of government or professional advisory committee; senior position in non-government breast screening organisation or consumer group. We sought to maximise the diversity of perspectives amongst our participants by deliberately seeking experts known to have publically expressed divergent opinions about breast screening (loosely categorised by us as being “supportive”, “mostly supportive” or “critical”) and experts from a range of professional roles across Australia including clinical practice, research, program administration, advisory staff and consumer advocacy.

We identified potential participants by reading local academic and lay literature; scanning personnel lists on websites of government and non-government organisations; and following up on suggestions from previously interviewed experts and from colleagues involved in cancer screening research. We approached 46 experts via email and interviewed 33 (17 males, 16 females). The remainder were unavailable (1), unwilling (3) or did not respond (9). We had a particularly low response rate from volunteers in consumer advocacy roles, which may have been at least partly due to a higher turnover of people in these positions than in other professional roles – that is, they may no longer have been acting in a senior advocacy capacity when our email was sent.

We performed our analysis in parallel with data collection, and used the information in the early interviews to direct further sampling, aiming to capture and explore the range of different ideas about this topic. We continued sampling until we were satisfied that we had sufficient diversity of opinions and roles [58] (Table 1) and until we were no longer hearing any new information. (thematic saturation) [54, 57].

**Table 1:** Characteristics of experts

	Participants 33 (Experts who were invited but did not-participate 13)	
Professional role^a^	Clinicians^b^15 (3)	Oncologists 3 (1)
		Surgeons 4 (0)
		Breast physicians 1 (2)
		Radiologists 2 (0)
		Radiation oncologists 2 (0)
		Pathologists 3 (0)
		Other 0 (1)
	Non-clinical researchers 14 (3)	Epidemiologists/biostatisticians 9 (1)
		Other 5 (1)
	Administrators/managers 6 (2)	6 (2)
	Advocacy leaders 6 (7)	Consumers working in advocacy 3 (6)
		Clinicians/researchers working in advocacy 3 (1)
Public stance on breast screening^c^	Supportive 16 (9)	
	Mostly supportive^d^ 3 (1)	
	Critical 6 (0)	
	Unknown to researchers 8 (3)	

^a^note that some experts held more than one professional role, for this reason the numbers attached to specific professional roles do not neatly add up to *n* = 33 (participants) or *n = 13 (experts invited but not participating)*

^b^most clinicians engaged in research to a greater or lesser extent

^c^We loosely categorised potential interviewees as being “supportive”, “mostly supportive” or “critical” about breast screening based on publicly available commentary

^d^broadly supportive of breast screening but with selected concerns about one or more elements of the program

### Data collection

LP conducted semi structured interviews between October 2012 and October 2013. The interviews lasted an average of 66 min (range 39–105 min) and were conducted in the expert’s or in LP’s workplace, or by telephone if unavailable to meet in person. Making use of telephone interviews enabled us to speak with experts from disparate locations around the country and we found that telephone interviews were similar in quality and length to face-to-face interviews [59].

LP discussed her interest in the topic with experts, explaining that she was a medical practitioner with clinical experience in breast screening, currently undertaking doctoral studies in cancer-screening ethics. She informed participants that the purpose of the interviews was to glean the range of opinions amongst Australian experts about breast screening. They were asked about their general attitudes to the current program, their suggestions and hopes for the future of the program, and their opinions on communicating with consumers (Additional file 1). The interviews were digitally recorded, professionally transcribed and any identifying information (such as person or place names) that was articulated during the discussion was removed from the transcripts before analysis began.

### Analysis

Analysis involved iterative reading, coding and categorisation of interview data. We sought to identify and understand the range of attitudes and underlying values that experts expressed around the topic of consumer communication. Repeated reading was undertaken in conjunction with the generation of a set of codes that captured attitudes and values, and the development of more abstract categories, that evolved as data collection and analysis progressed. LP wrote case-based memos throughout the project and shared these and provisional analysis with the other authors [58]. All authors contributed to ongoing analysis, involving comparison between codes and data, revision of findings and development of concepts presented in this paper.

### Ethics approval

Ethics approval was granted from the Cancer Institute NSW Population & Health Services Research Ethics Committee [HREC/12/CIPHS/46] and the University of Sydney Human Research Ethics Committee [#15245]. All participants gave written or verbal informed consent to their involvement in the study (those who were interviewed face to face gave verbal consent; those who were interviewed via telephone gave verbal consent). This research complies with current Australian laws and guidelines.

## Results

### Expert opinions on communicating with consumers

Experts spoke in detail about communicating with consumers regarding breast screening. Their comments focused on two issues: 1) the degree of guidance for consumers, and 2) the extent of information provided to consumers about overdiagnosis. Table 2 shows how experts’ views on communication could be divided into three approaches according to the interaction between these two issues (guidance and overdiagnosis information). The first approach, which we have named “Be screened”, combined guiding consumers towards screening with limited information on overdiagnosis. The second approach, “Be screened and here’s why”, combined guiding consumers towards screening with full consumer information. The third approach, “Screening is available, please consider whether it’s right for you” combined no guidance about screening with full consumer information. We found a similar level of expert support for each of the three approaches. Logically there could potentially have been a fourth approach (no guidance and limited information – see Table 2) but there were no experts who advocated for this position. All experts were in favour of either guidance or full information or both; there were no experts who would recommend no guidance and no means for consumers to make an informed choice of their own.

**Table 2:** Experts’ preferences regarding guidance and information when communicating with women about breast cancer screening

	Guidance^a^	No guidance^b^
Limited information^c^	*“BE SCREENED”* ^*e*^	*N/A*
Full information^d^	*“BE SCREENED AND HERE’S WHY”* ^*e*^	*“SCREENING IS AVAILABLE, PLEASE CONSIDER WHETHER IT IS RIGHT FOR YOU”* ^*e*^

^a^Guidance: Experts who preferred guiding women to screen advocated for the following:

• Provider-to-consumer guidance to screen via public promotional advertising & personalised letters of invitation to women from the screening program

• Marketing support & participation targets for local breast screening units to ensure guidance is effective at maintaining high participation

• Educational support & electronic reminders to enhance GP-to-consumer guidance to screen

^b^No guidance: Experts who preferred not to guide women’s screening choices advocated for the following:

• An independent body to provide information to women about screening options & encourage them to make a thoughtful choice about participation

• Online decision aid tools available to consumers

• No personalised invitations

• Targets for consumer understanding rather than participation

• Educational support to enhance GP assistance for women to make an informed screening choice

• Directed advice available from GP upon request

^c^Limited information: Experts who preferred limiting the overdiagnosis information presented to women advocated for the following content in consumer communications:

• Information that breast screening saves lives

• Information that a recall does not necessarily mean you have cancer

• Brief mention that overdiagnosis is a possibility and that it is unlikely

• Advising that further information is available to women upon request

^d^Full information: Experts who preferred providing full information to women advocated for the following content in consumer communications:

• Detailed information about mortality benefit, false positives & overdiagnosis associated with breast screening

• Numerical / pictorial comparison of chances of deriving benefit & being overdiagnosed

^e^There were roughly equal numbers of experts supporting each of the three named approaches

Overall more experts preferred guiding women to be screened, and overall more experts preferred that full information be provided. Examining Table 2, and recalling that there were approximately the same number of experts in each cell, reveals why. Two out of three approaches (“Be screened” and “Be screened and here’s why”) supported guidance to screen. Two out of three approaches (“Be screened and here’s why” and “Screening is available”) supported the provision of full information on overdiagnosis. Thus providing guidance, and providing full information, were preferred to the alternatives.

### Expert descriptions of what it means to guide consumers to be screened, or not

The detail in Table 2 describes experts’ ideas of what it means to guide consumers to be screened, or not. The majority argued that consumer communication should include guidance towards screening. They endorsed the existing strategy whereby the screening provider is the main source of guidance. They also approved of current participation targets for screening units, suggesting they were a useful tool for developing and maintaining a successful guidance strategy. Several experts advocated extending and enhancing consumer guidance by providing greater marketing support to local screening units, along with education and reminders for primary care practitioners to take a more active role in promoting breast screening.

A smaller number of experts recommended against guiding women to participate in screening (Table 2). These experts suggested that consumers be educated about the availability of screening, encouraged to understand benefits and harms, then asked to carefully consider whether or not the program was right for them. They recommended that communications with consumers be written by an independent body, suggesting that providers were likely to view and/or present screening in a favourable light. These experts were in favour of replacing participation targets with targets around information provision or public understanding of screening. They opposed personalised letters of invitation, suggesting that these carried the weight of government support and would therefore be seen by women as persuasive, even coercive. A couple of experts explicitly suggested that women should be given assistance with decision making, discussing strategies such as online decision making tools and primary care practitioner support in understanding the evidence and making choices in accordance with consumers’ personal values. They suggested that guidance about screening could be made available for those who wanted it.

### Expert descriptions of what it means to inform consumers about overdiagnosis, or not

Experts described two approaches to information about overdiagnosis (Table 2). The majority of experts thought that information about overdiagnosis should be limited. These experts thought consumer communications should impart simple, uncomplicated information about screening benefit, with limited detail on possible downsides. They suggested overdiagnosis information should be presented briefly along the lines of, *“some of the things that we are going to be treating you for may not progress.”* (Expert #33, clinician and provider) These experts proposed that further information could be made available for those who wanted it. Contrary to this position, a smaller group of experts advocated full information about both benefits and harms of breast screening. They particularly wanted consumers to be provided with understandable data about overdiagnosis, including readily comparable information on chances of mortality benefit versus overdiagnosis.

### Experts’ reasoning about their preferred communication approach

Experts gave a variety of reasons to explain their positions on communicating with consumers. Table 3 presents the range of reasons for experts’ preferred approaches to breast screening communications. Further data, including quotations from experts that encapsulates the range of reasoning about communications, is included in Additional file 2. The major concerns of experts are discussed below.

**Table 3:** Experts’ rationales for their stance on guidance and information provision to women regarding breast screening

Guiding women towards breast screening
FOR
● Maximises screening participation^a^
● Saves lives^a^
● Women will have more treatment options^a^
● Overall, screening delivers more benefits than harms to the population^a^
● Overdiagnosis is not a harm
● Providing guidance about good health is a government public health responsibility
● You don’t want people to make decisions in public health, you just want them to follow advice
● Expecting consumers to make their own informed choice is unfair and unrealistic because the evidence is so complicated
● (Some) people want to be told what to do
AGAINST
● Individuals should be free to make their own decisions^a^
● Personal autonomy is important^a^
● Harm:benefit ratio is equivocal so screening should be an individual choice, not a government-promoted activity^a^
● Screening affects only the individual concerned, so there is no community-benefit argument to justify promotion of screening
● Others may not have the best interests of the individual consumer at heart
● Consumers tend to be better than policy makers at remembering to consider screening harms as well as benefits, so judgements about screening should be left to consumers
● The harms of breast screening are greater than the benefits
**Limiting consumer information on overdiagnosis**
FOR
● Maximises screening participation^a^
● Calling overdiagnosis a “harm” is just one (mis)interpretation of the facts
● Women don’t consider overdiagnosis a harm; main harms that women care about are: pain, hassles of parking and making appointments, radiation, breast damage, anxiety about recalls
● Population based information on overdiagnosis is not applicable to individuals
● The real problem is not overdiagnosis but overtreatment
AGAINST
● People should know what they are signing up for when they participate in screening^a^
● Providing information enables informed decision making^a^
● Informed decision making is particularly important for breast screening because there are some downsides^a^
● Providing full information is a professional responsibility
● (Some) women want full information

^a^very strongly/frequently expressed reasons

### Experts’ reasoning about guidance to attend screening

Experts who preferred guidance for consumers were particularly concerned to maximise screening participation rates in order to deliver breast cancer related benefits to individuals and populations. Many also reasoned that guidance was important because benefits of screening outweighed harms. Some experts added to this by asserting that overdiagnosis was not a harm, rather that the diagnosis of small cancers was exactly what the screening program was intended to do in order to reduce breast cancer mortality and morbidity. Experts also argued beyond the breast screening context, suggesting that providing advice and guidance on health matters were important public health responsibilities.

Experts who advocated against guidance were worried about overdiagnosis harms and were enthusiastic about enabling individual consumers to make their own decisions about health. They suggested that independent consumer decision making was particularly important in breast screening because of the close balance between benefits and harms, and what experts saw as the individual nature of the benefits. These experts suggested that, unlike some other public health programs, there was no community benefit associated with individual participation in breast screening: *“there is a community benefit from immunisation, but there’s no such community benefit from screening. Like, the benefit is to the individual,”* (expert #8, researcher) because, “*if I choose not to go, the only person that’s being harmed by my choice is me. I’m not giving the person next door to me breast cancer.”* (Expert #27, researcher). They also expressed concerns that breast screening enthusiasts might not necessarily act in the best interests of individual consumers. For example, these experts suggested that governments might be driven by the promise of political gain from addressing women’s health, and that providers and clinicians may have vested interests in their own employment security and remuneration.

Both groups referred to evidence-based decision making to justify their positions about guidance. Those preferring guidance suggested that individual consumers would be unable to understand the complex evidence and should therefore be provided with advice from experts about where the balance of benefits and harms lies. Those against guidance suggested that consumers, rather than experts, were better placed to use the evidence appropriately, since experts tended to ignore the harms and focus on the benefits. One expert advocated against guidance on the basis that, as they saw it, the evidence showed breast screening was likely to deliver more population harms than benefits. They believed that advising against screening was politically unacceptable, so removing guidance to screen was the next best option.

### Experts’ reasoning about providing information on overdiagnosis

Experts who expressed a preference for limiting information to consumers were mainly concerned about the potential impact of discussing overdiagnosis. They suggested that detailed information about overdiagnosis may result in consumers becoming confused or scared, decreasing the likelihood that they would attend screening, and reducing their options for life-saving treatment. As noted above and in Table 3, many of these experts challenged the conception of overdiagnosis as a harm, and used this to justify their support for both guidance and limited information. Importantly, these experts did not see their preference for limiting overdiagnosis information as being against informed decision making. Many of these experts were consumer advocates, and were strongly supportive of informed patient choice in relation to breast cancer treatment. They explained their apparently contradictory position on information about screening versus treatment in two ways. Some stated that the concept of overdiagnosis being a harm was based on opinion, rather than fact and therefore did not count as information. Other experts in this group suggested that maximising screening uptake would enhance patient choice (about life-saving treatments) because early knowledge of breast cancer status was an important part of this.

Experts who preferred full information argued that consumers should be informed about what they were being asked to do. In particular these experts claimed that full information on overdiagnosis was important for its instrumental role in informed consumer decision making.

### Experts’ reasoning was grounded in underlying values

Table 4 shows how experts prioritise and conceptualise values differently when discussing their communication preferences. Some experts explicitly referred to values, naming principles such as “delivering benefits”, “respect for autonomy” and “avoiding harms”. Other experts were more concrete in their discussion, but underlying values could be readily discerned. Abstracting the experts’ reasoning in this way clarifies how values were used and prioritised in association with particular communication preferences. For example, experts who advocated for “Be screened” prioritised the values of delivering benefits and delivering more benefits than harms. Those who recommended “Be screened and here’s why” added transparency to this list. Experts who advocated for “Screening is available” prioritised avoiding harm, delivering more benefits than harms and respect for autonomy.

**Table 4:** Experts’ conceptualisation and prioritisation of values in three approaches to communication with consumers

Values	Conception of values underpinning the “Be screened” approach	Conception of values underpinning the “Be screened and here’s why” approach	Conception of values underpinning the “Screening is available, please consider whether it is right for you” approach
Delivering benefits	Reduced breast cancer mortality & reduced treatment related morbidity^a^	Reduced breast cancer mortality & reduced treatment related morbidity^a^	Reduced all cause mortality and morbidity
Avoiding harm	Minimising pain, parking hassles, radiation, anxiety about false positives	Minimising pain, parking hassles, radiation, anxiety about false positives	Minimising overdiagnosis harms^a^
Delivering more benefits than harms	Experts informed by evidence to assess population benefits & harms^a^	Experts informed by evidence to assess population benefits & harms^a^	Consumers informed by evidence and personal values to assess balance of benefits & harms for themselves^a^
Respect for autonomy	Maximising consumer choices for life saving breast cancer treatment; freedom from misleading influences on consumer screening participation	Maximising consumer choices for life saving breast cancer treatment	Facilitating informed consumer decision making about screening, freedom from external (positive or negative) influences on decision making^a^
Transparency	n/a	Telling consumers what might happen when participating in screening^a^	Telling consumers what might happen when participating in screening
Professional responsibility	Providing guidance on healthy living to the population	Providing guidance on healthy living to the population	Providing full information to the population about healthy living
Respect for public preferences regarding decision-making responsibility	Consumers want to be told what to do	Consumers want to be told what to do; consumers want full information	Consumers want full information

^a^values that were prioritised for this particular communication approach

Table 4 also shows that experts conceived of or applied values differently, such that the same abstract value was sometimes used to justify opposing communication preferences. For example, although respect for autonomy was prioritised by some more than others, all experts were able to use this value to justify their preferences. Those experts who preferred “Be screened” and “Be screened and here’s why” saw the provision of guidance to screen as being respectful of autonomy because it would maximise consumer choices around breast cancer treatment. Those who preferred the “Screening is available” approach suggested a no guidance agenda would better respect autonomy because it facilitated informed consumer decision making without expert or governmental influences.

## Discussion

This study is, we believe, the first empirical exploration of experts’ views on communicating with consumers about breast screening. We found experts considered the most important elements of this communication were the degree of guidance and the amount of information on overdiagnosis. These interacted to produce three approaches to consumer communication: “Be screened”, “Be screened and here’s why” or “Screening is available, please consider whether it is right for you”. We expected that experts would be conversant with academic and public debates, and our study confirms that their views on breast screening communications reflect ideas being discussed in the literature [45]. The existence of controversy about breast screening is widely recognised; our results deliver both empirical confirmation and practical detail to this broad recognition.

Our study explored the reasoning and motivation of experts. Our analysis fits with and builds upon what others have suggested about the aims of screening communication. Many writers discuss what they see as the competing goals of maximising participation versus respecting consumer autonomy by facilitating informed choice about screening [29, 36, 60]. Our study explains the reasoning of experts who aim to achieve one or both goals. The detail in our study provides some insights into why debates about communication persist. We found experts disagree on what values to prioritise when considering communication strategies and have different conceptions of what it means to respect a particular value, such as autonomy, in the context of breast screening. These results validate previous, more theoretical, discussions about possible variations in use and conception of values in healthcare [16, 61, 62] and extend other research looking at experts’ values in breast screening generally [52]. It is not only the values of experts that are important of course, but also the views and attitudes of the public: our study sits alongside and complements ongoing work into ascertaining public opinion about topics such as consumer communication on cancer screening [63, 64].

This study has implications for current debates about the use of ethics frameworks in public health. The Four Principles approach to medical ethics [65] is well recognised as a useful tool for assisting decision making in clinical practice and there is ongoing interest in promoting ethical care alongside or as part of evidence based medicine [66]. There is increasing recognition that the particular aims, responsibilities and challenges of public health as distinct from clinical medicine might be better served with a specific set of principles or values [67, 68]. While there is ongoing discussion about what this might look like, there seems to be broad support for some kind of values-based public health ethics framework. Our study, however, illustrates the complexity of using such an apparently simple framework in a particular, practical context, by showing that the prioritisation and interpretation of the same values amongst influential experts is not consistent. Significantly, our results indicate that the same bare list of values could be used by different experts to potentially justify each of three very different communication approaches. In order to use values and principles to assist and steer policy, rather than “rubber stamp” existing plans, greater discussion of the meanings of values is required, situated in a concrete context (in this case, breast screening).

To support experts and others who are involved in shaping polices on communication with consumers about breast screening, we suggest the following questions as a structure to guide decision-making:

### What values should drive this communication?

A wide range of values relevant to public health should be considered before deciding which one/s should be prioritised in this particular context. Those involved in discussions might start with the values that have been discussed in this paper: delivering benefits, avoiding harms, delivering more benefits than harms, respect for autonomy, transparency, professional responsibility and respect for public preferences regarding decision-making responsibility. The ethics literature suggests other values that were not raised by these experts, including distributive justice, procedural justice and trust [67–70]. Deciding which of these values to prioritise in any given context will not always be easy [68]. For the purposes of communication with consumers about breast screening, there is likely to be strong debate around the relative importance of two potentially conflicted values: delivering more benefits than harms, and respecting autonomy. Central tasks here are to agree which values are important and develop a shared understanding of what these values mean [52]. Existing public health ethics frameworks provide some guidance [67–70].

### How will selected value/s be prioritised?

In order to address this question it may be useful to debate different conceptions of values and consider what communication aims would correspond with each. Note that the priority value/s are decided first, and these will help to identify and guide the stated aims of the communication. Imagine, for example, the main value is to deliver more benefits than harms. This raises the question of whether it should be experts or consumers who define which benefits and harms matter and how they are weighed. If the conclusion is that experts should decide, then the aim of communication may be to persuade consumers to act in line with expert assessment. If, in contrast, the decision is that consumers should make their own decisions about which benefits and harms matter and how they should be weighed, then the aim of screening communication would be to encourage consumer understanding and choice.

### What communication strategy corresponds to these selected aims?

It will be necessary at this point to build on answers to the questions above. For example, if it is decided that the main value is to deliver more benefits than harms, and that this is best achieved by persuading consumers to act in accordance with expert opinion, then a “Be Screened” approach would be recommended (or a “Be Screened and here’s why” approach, if transparency was also selected as an important value). However if it is decided that delivering more benefits than harms is best achieved by encouraging consumer understanding and choice then the “Screening is available, please consider whether its right for you” strategy will be selected for communicating about breast screening.

Our study’s strengths include its detail and depth of coverage through interviews with a broad range of experts from different fields and locations across the country.

We must consider that the study may be limited due to its geographic focus on Australian experts. It is likely, however, that our findings will have broader application beyond this country since the nature and detail of the breast screening program in Australia is similar to those throughout much of UK and Europe, and the values and principles discussed by the experts are well recognised worldwide. It is also possible that our findings are limited by the participating sample – that is, we must consider the question of whether or not the experts who were asked but did not participate held different views to those who did participate. Since we specifically sought to include participants from a range of professional roles and attitudes to screening, and since we continued sampling until we reached thematic saturation, we are confident that our study has mapped a sufficiently wide range of opinions and values [57].

## Conclusions

This study provides the first empirical explanation of why well-informed experts take such different views on communication with consumers about breast screening. Experts do not necessarily have the same values priorities in mind, and even if they do, they do not necessarily agree on what actions would be in line with that particular value. Thus there are layers of difficulties in implementing recommended public health ethics frameworks as guidance for public health policy. We advocate for greater research into values thinking amongst public health policy makers, and would encourage explicit and ongoing discussions about what values mean and which ones are important and why. In the meantime we provide step-by-step guidance as to how to use values in policy making within the context of breast screening in order to develop ethically robust communication strategies for consumers.

## Additional files

Additional file 1: **Sample interview questions.** (DOC 27 kb)
Additional file 2: **Figure 3  Experts’ rationales for their stance on guidance and information provision to women regarding breast screening (includes expert quotes).** (DOC 82 kb)

## Abbreviation

RCT: Randomised controlled trial
