# Supplementary material for: The role of communication in breast cancer screening: a qualitative study with Australian experts
Source: BMC Cancer. 2015 Oct 19;15:741. doi: 10.1186/s12885-015-1749-0 (PMC4617891; doi:10.1186/s12885-015-1749-0)
Supplement: Additional file 1: — Sample interview questions. (DOC 27 kb) [file 12885_2015_1749_MOESM1_ESM.doc]

**Additional file 1**

**The role of communication in breast cancer screening: a qualitative study with Australian experts**

Lisa M Parker, Lucie Rychetnik, Stacy M Carter

Corresponding author: Lisa M Parker

Centre for Values, Ethics and the Law in Medicine (VELiM),

Sydney School of Public Health,

The University of Sydney,

[lisa.parker@sydney.edu.au](mailto:lisa.parker@sydney.edu.au)

*Sample interview introduction and questions (note: this list is provided as a guide only; the questions were modified to suit the experience and perspective of the interviewee)*

Thank you for agreeing to participate in this study. As you know, there has been quite a lot written in the literature and in the media about breast screening and what the program should look like. Plenty of people are happy with things the way they are, but others are not. So I’m interested in exploring that range of opinion, particularly amongst people who work in the field, including those who work in clinical practice, research, administration, or in breast cancer advocacy.

- Can you describe the scope of your professional activities that involve breast screening, to give me an idea about your involvement in the program?
- Would you like to see any changes to the current program?
  - *Prompt*: What would your ideal program be?
- There are many different ideas about breast cancer screening. Can you comment on these?
  - *Prompt*: There are some who hold very extreme views about breast cancer screening. How do you respond to these ideas? What do you think drives those views?
- *(If the topic hasn’t yet surfaced)* One of the topics I’m interested in is communicating with women. You may know that some places are looking at re-doing the breast screening leaflet. What are your thoughts on what should be said to women?
