# Supplementary material for: The role of communication in breast cancer screening: a qualitative study with Australian experts
Source: BMC Cancer. 2015 Oct 19;15:741. doi: 10.1186/s12885-015-1749-0 (PMC4617891; doi:10.1186/s12885-015-1749-0)
Supplement: Additional file 2: — Figure 3 Experts’ rationales for their stance on guidance and information provision to women regarding breast screening (includes expert quotes). (DOC 82 kb) [file 12885_2015_1749_MOESM2_ESM.doc]

**Additional file 2**

**The role of communication in breast cancer screening: a qualitative study with Australian experts**

Lisa M Parker, Lucie Rychetnik, Stacy M Carter

Corresponding author: Lisa M Parker

Centre for Values, Ethics and the Law in Medicine (VELiM),

Sydney School of Public Health,

The University of Sydney,

[lisa.parker@sydney.edu.au](mailto:lisa.parker@sydney.edu.au)

**Figure 3 Experts’ rationales for their stance on guidance and information provision to women regarding breast screening (includes expert quotes)**

| **Guiding women towards breast screening**  **FOR** | |
| --- | --- |
| Maximises screening participation^a^ | *“The key thing is getting women in to screen and getting them there to be screened”* (Expert #31, consumer advocate) |
| Saves lives and means that women have more treatment options^a^ | *“Early detection is very important in terms of the treatment options that are possible for the women. For example smaller tumours are likely to result in less invasive surgery, less radical surgery. So a lumpectomy, verses a mastectomy. Early detection is thought to be also important in survival outcomes too, but that’s obviously been a mixture of early detection plus improved treatment.”* (Expert #6, consumer advocate)  *“These patients with clinically palpable lesions may then not be conservable; they may only have an option of mastectomy. They may have had an option of chemotherapy or not, whereas they’ll all be looking at chemotherapy if it’s progressed and therefore also radiotherapy”* (Expert #3, clinician) |
| Overall screening delivers more benefits than harms to the population^a^ | *“The whole premise of population improvements are that some people are not going to benefit at all from the intervention. And so if you take a vaccine, if you take - you take anything, some people will react, you know. But on average you're hoping to do better …* [With breast screening] *look at the benefits of the intervention …* [and] *the costs of it. And there is no easy ethical call on that… by being part of this program, it accepts that some people at high risk are going to go on a more rapid trajectory for treatment, and some of them who would have just become normal get intervened upon and they have psychosocial consequences as a result of it.”* (Expert #26, epidemiologist) |
| Overdiagnosis is not a harm | *“Harm is a term that’s been developed by academics, along academic lines…* [Overdiagnosis is not] *women’s definition of harm” (*Expert #24, consumer advocate) |
| Providing guidance about good health is a government responsibility | *“By the time the governments have accepted that it’s a good thing, I think the government’s role is just to go all-out advertising it positively”* (Expert #7, clinician) |
| You don’t want people to make decisions in public health, you just want them to follow advice | *“There’s this idea that everybody has to go through individual decision making. Whereas, see, in public health, you don’t want people to make individual decisions about washing their hands or getting their children immunised. You just want them to do it… Now this might sound a bit extraordinary but it’s not really, for public health people”* (Expert #10, epidemiologist) |
| Expecting consumers to make their own informed choice is unfair and unrealistic because the evidence is so complicated | *“When people argue a lot, you know, people that are informed, supposedly, argue, I don’t know how* [women] *give informed consent. It’s very difficult for the average layperson to understand”* (Expert #11, clinician)  *“Explaining the odds … is hard and you can put little figurines in this box, with - so many women might, if you’re in this box, you might be there, but nothing is so black and white and everything is kind of nuanced. And it’s really hard to see people making judgements independent of what the doctor might think is the best route possible.”* (Expert #26 epidemiologist) |
| (Some) people want to be told what to do | *“Some people don’t want to be involved; some people just want to be told what to do.”* (Expert #24, consumer advocate) |

| **Guiding women towards screening**  **AGAINST** | |
| --- | --- |
| We should allow individuals to make their own decisions^a^ | *“We can’t … be like grandparents and say, ‘You have got to do this,’ and impose our will on them.”* (Expert #33, clinician & provider) |
| Personal autonomy is important^a^ | *“We just, I don’t think, want to have bodies like governmental bodies or any other sorts of bodies making decisions about what people ought and ought not to be doing because it’s good for them… I think we have to respect people’s autonomy, I think it’s a basic principal in the democracy and I think you have to respect it.”* (Expert #27, epidemiologist) |
| Population benefits and harms are finely balanced and thus consumer attitude to risk is relevant to likelihood of delivering more benefits than harms to the individual^a^ | *“There’s actually a lot of evidence of harm here. If I look at it carefully, then you think oh, the benefits and the harms are much more finely balanced than I had actually appreciated, then a persuasion campaign is just indefensible.”* (Expert #27, epidemiologist) |
| There is no community benefit or harm attached to participating in screening | *“The only person who’s going to be harmed* [if they don’t attend screening] *is the person themselves – I mean, probably and their families because of the consequences of the treatment – but I might apply a different level of – a different sort of standard to that compared with, say, something like immunisation where people’s decision not to be immunised affects people other than themselves.”* Expert #21, epidemiologist)  *“If I choose not to go, the only person that’s being harmed by my choice is me. I’m not giving the person next door to me breast cancer… whether I go or not doesn’t mean that anybody else is more or likely to get breast cancer. So I don’t think persuasion or enforcement has a role there.”* (Expert #27, epidemiologist) |
| Others may not have the best interests of the individual consumer at heart | *“You’ll get politicians like Bob Hawke who think that adding the screening program in will buy them votes … the consultants, who are often people with vested interests, like the radiologists or the breast cancer surgeons.”* (Expert #8, researcher) |
| Consumers are better at considering both benefits and harms rather than just focusing on the benefits | *“At the public policy level and at that community emotive level, there is a tendency to ignore cost and harm and focus on the benefits. At the personal level … it looks like women can* [better] *understand that argument about overdiagnosis.”* (Expert #20, epidemiologist) |
| The harms of breast screening are greater than the benefits | *“The evidence in Australia is that the health benefit : harm ratio is simply too high … opportunity costs are too high… I would argue very strongly that* [instead of breast screening] *we increase our emphasis on getting women to present … early* *… and make sure that the healthcare system can diagnose disease competently in women with symptoms and treat them optimally …* *My strategy for unwinding* [the breast screening program] *would be to stop the invitation and provide the information”* (Expert #12, clinician) |

| **Limiting consumer information on overdiagnosis**  **FOR** | |
| --- | --- |
| Maximises screening participation^a^ | *“To give them that much information I think would scare them. They’d chuck it in the rubbish, and they’d be, like, this is too hard”* (Expert #25, provider) |
| Calling overdiagnosis a “harm” is just one (mis)interpretation of the facts | “[Information on overdiagnosis] *is a bit of a worry, because of the way it’s presented and interpreted … Women aren’t being harmed by breast screening and society isn’t being harmed by breast screening… It comes from the epidemiologists, who are quite far removed from actually having breast cancer or treating it - they’re looking at populations and then they take it upon themselves … to actually put their personal view as to what this might be doing to women, what harm it might be doing, which is very unscientific. Early diagnosis, breast screening, leads to more of your diagnoses and overtreatment and that’s* [not] *a harm, it’s a value…* *That bit of information* [about the harms of overdiagnosis] *is opinion interpretation… You have to be very careful what information you do give* [women] *and that you’re not giving them a set of facts that’s been interpreted by one kind of view* (Expert #13, consumer advocate) |
| Women don’t consider overdiagnosis a harm; main harms that women care about are: pain, hassles of parking and making appointments, radiation, breast damage, anxiety about recalls | *“Pain and parking, right? They were the two complaints that women had about mammograms. And also having to book.”* (Expert #10, epidemiologist)  *“If you ask women what harm is from breast screen they’ll say, maybe, harm is the x-rays that you get. Maybe harm is the fact that you have your breast squeezed so much and that might cause damage to your breasts … As long as you’re given the key factors … you know, ‘We just thought this is your first time at Breast Screen, you better come back for further diagnostic images’* [so] *then if you are going to be recalled then you’re not panicked.’* (Expert #24, consumer advocate) |
| Population based information on overdiagnosis is not applicable to individuals | *“I don’t think you need to put in the business about overdiagnosis ‘cause you don’t actually know that it’s overdiagnosis. Until you know which women were – that some were actually – you don’t know. It’s just not there, so I don’t think it’s very straight reasoning, to get those very big picture things and try to apply them to the individual, when you don’t know whether they could possibly apply to the individual.”* (Expert #13, consumer advocate) |
| The real problem is not overdiagnosis but overtreatment | *“I always like to think* [the harm] *is not necessarily overdiagnosis but overtreatment … So don’t stop yourself from actually being diagnosed but then when you get the information, it’s what you do with it. And the patient needs to be very well informed around what your risks are if there’s no treatment versus the treatment* (Expert #6, consumer advocate) |

| **Limiting consumer information on overdiagnosis**  **AGAINST** | |
| --- | --- |
| People should know what they are signing up for when they participate in screening^a^ | *“They need to be provided with adequate information to know what they are signing up for. If they are coming to screening they need to know.”* (Expert #33, clinician & provider) |
| Providing information enables informed decision making^a^ | “[We should let] *women know in an intelligent way about this complex topic so that they can be fully informed and make an informed choice … you don’t want to just say, trust me I’m a doctor”* (Expert #22, clinician & provider) |
| Informed decision making is important because there are some downsides to breast screening^a^ | *“This is not straightforwardly a good thing. There are some downsides and while we don’t necessarily think the downsides are such that you shouldn’t be doing it, at the very least, we should be telling women about this so that they can make an informed decision.”* (Expert #20, epidemiologist) |
| Providing full information is a professional responsibility | *“We have a responsibility for them to understand why they’re coming”* (Expert #4, clinician)  *“I do believe … that people working in public health have a responsibility to talk about their work and to educate the community. I don’t think it’s something where you do the work, you publish it in an academic journal and that’s the end of it.”* (Expert #21, epidemiologist) |
| (Some) women want full information | *“People ... are different in … their needs for information … you have to cover the high information needs people”* (Expert #23, clinician) |

^a^ Very strongly / frequently expressed reasons
